# Validity and reliability of the short form-late life function and disability instrument Turkish version

**DOI:** 10.1371/journal.pone.0361134

**Published:** 2026-11-10

**Authors:** Mert Ataş, Sevil Ceyhan Doğan

**Affiliations:** 1 Sivas Numune Hospital, Physical Medicine and Rehabilitation Department, Sivas, Turkey; 2 Sivas Cumhuriyet University Faculty of Medicine, Physical Medicine and Rehabilitation Department, Sivas, Turkey; Gelisim University: Istanbul Gelisim Universitesi, TÜRKIYE

## Abstract

The growing aging population worldwide has increased the need for valid, culturally adapted tools to assess function and disability in older adults. The Short Form–Late Life Function and Disability Instrument (SF-LLFDI) is a brief, psychometrically sound tool originally developed to evaluate physical functioning and disability in community-dwelling older adults; however, no Turkish-validated version existed prior to this study. This study aimed to translate, culturally adapt, and evaluate the psychometric properties of the Turkish SF-LLFDI. Cross-cultural adaptation followed Beaton et al.‘s internationally recognized guidelines, involving forward translation, back-translation, expert committee review, and pilot testing with 25 participants. A total of 230 community-dwelling adults aged ≥70 years were recruited. Reliability was assessed via internal consistency (Cronbach’s α) and test-retest stability using the Intraclass Correlation Coefficient (ICC) in a subsample of 77 participants over a 15-day interval. Construct validity was examined through exploratory (EFA) and confirmatory factor analyses (CFA), and criterion validity was evaluated by correlating SF-LLFDI scores with the Barthel Index, Lawton-Brody IADL, Short Physical Performance Battery (SPPB), Timed Up and Go Test (TUG), SF-36, Mini Mental State Examination (MMSE), and Charlson Comorbidity Index. The Turkish SF-LLFDI demonstrated excellent internal consistency (α = 0.987) and outstanding test-retest reliability (ICC = 0.997–0.999). EFA confirmed the original factor structure, accounting for 83.13% of the variance in the Function subscale and 79.83–85.00% in the Disability subscales. CFA indicated adequate model fit (χ^2^/df = 2.76–4.71, RMSEA = 0.069–0.078, CFI = 0.960–0.984, GFI = 0.900–0.947). Strong concurrent validity was evidenced by significant correlations with the Barthel Index (r = 0.960), Lawton-Brody IADL (r = 0.937), SPPB (r = 0.986), TUG (r = −0.975), MMSE (r = 0.848), SF-36 (r = 0.664–0.938), and Charlson Comorbidity Index (r = −0.897) (all p < 0.001). The Turkish SF-LLFDI is a valid and reliable instrument for assessing function and disability in Turkish-speaking older adults, suitable for use in both clinical and research settings.

## Introduction

The increase in the elderly population worldwide has made identifying health-related outcomes associated with aging an important public health goal. The World Health Organization (WHO) predicts that by 2050, approximately 22% of the world’s population will consist of individuals aged 60 and over [1]. When evaluated specifically for Turkey, it is projected that by 2040, the proportion of individuals aged 65 and over in the total population will exceed 16% [2]. This expected change in the societal structure will also bring about changes in public health approaches and will make evaluating the functionality and disability status of the elderly population an important objective [3]. Among the most commonly used scales for the assessment of disability and functionality are the Barthel Index of Activities of Daily Living [4,5] and the Lawton-Brody Instrumental Activities of Daily Living Scale [6,7]. However, these scales do not incorporate a disability model and therefore lack the capacity to assess components of disability such as social participation and environmental factors. Therefore, there is a need for a measurement tool based on a conceptual model of disability, which has a high capacity to evaluate social participation and environmental factors, to be used in the elderly population. To address this need, the Late Life Function and Disability Instrument (LLFDI) was developed by Alan Jette et al.; it is based on the widely used Nagi and ICF models for assessing disability and functionality [8]. Because it is based on conceptual disability models, the LLFDI can be used as a guide in the development of specific rehabilitation programs that encompass physical factors, social factors, and environmental factors [9,10]. LLFDI consists of functionality and disability subscales and includes a total of 48 questions, which can be completed in approximately 30 minutes [11].

In a study conducted in 2009, a short form of the scale was created and its validity and reliability were demonstrated. With this form, named Short Form-Late Life Function and Disability Instrument, the evaluation time has been reduced to around 8 minutes, its applicability in different settings has increased, and it has become more practical [11]. Validity and reliability studies of the LLFDI have been conducted in Turkish [12], Swedish [13], Hebrew [14], Korean [15], Dutch [16] and Arabic [17], while validity and reliability studies of the SF-LLFDI have been conducted in Spanish [18] and German [19]. Validity studies have been conducted for COPD patients [20], heart failure patients [21], individuals with multiple chronic diseases [22], stroke patients [23], patients with neuromuscular disorders [24], and wheelchair users [25]. There is a need for a scale based on a conceptual model, comprehensive, and practical for use in Turkish-speaking populations. The aim of this study is to adapt SF-LLFDI into Turkish and evaluate the psychometric properties of the adapted version.

## Materials and methods

This methodological study was conducted in the Department of Physical Medicine and Rehabilitation at Sivas Cumhuriyet University. It aimed to adapt the Short Form Late Life Function and Disability Instrument (SF-LLFDI) into Turkish and to examine its validity and reliability in community-dwelling older adults. All participants submitted informed written consent before enrolling in the study, based on the ethical guidelines of the 2008 Declaration of Helsinki. Ethical approval was obtained from the Non-Interventional Clinical Research Ethics Committee of Sivas Cumhuriyet University (Approval No: 2023-12/42). Written permission to adapt the original scale into Turkish was obtained from Alan M. Jette, the original developer of the LLFDI, via email correspondence in November 2023. Participants were recruited between 01/01/2024 and 30/12/2024.

### Translation and cultural adaptation

The scale’s cross-cultural adaptation process was executed in accordance with the principles established by Beaton et al [26]. Initially, two separate forward translations into Turkish were conducted by bilingual physicians whose native language is Turkish.

The translations were amalgamated into a singular preliminary version. Subsequently, this synthesis version was translated back into English by three faculty members from the Department of English Language and Literature, who were unaware of the original version. A specialized committee, consisting of faculty from the Departments of Physical Medicine and Rehabilitation and English Language and Literature, evaluated all iterations to guarantee semantic, idiomatic, experiential, and conceptual congruence between the source and target versions.

A pilot test of the pre-final version was conducted with 25 participants. Completion of the questionnaire required approximately 25–30 minutes. Following completion of the questionnaire, participants were asked whether any item was difficult to understand, unclear, or culturally inappropriate. All participants reported that the items were clear and comprehensible, and no concerns, hesitation, or suggestions requiring modification were expressed. Therefore, no modifications were made to the translated version following the pilot test.

### Participants

A total of 230 community-dwelling adults aged 70 years or older were recruited. Participants were included if they: (1) were aged 70 or older; (2) had a Mini Mental State Examination (MMSE) score >24; and (3) were able to walk independently or with assistive devices. Exclusion criteria included: severe cognitive impairment, significant visual deficits, neurological or orthopedic disorders limiting participation (excluded to ensure that observed functional limitations reflected disability status rather than acute illness or injury), and failure to provide informed consent. The required sample size was calculated using the G*Power 3.1.9.7 software (Heinrich-Heine-Universität Düsseldorf, Düsseldorf, Germany) [27]. An a priori power analysis was performed using the Exact test family for a two-tailed correlation analysis under the bivariate normal model. Based on a significance level of α = 0.05, and an expected correlation coefficient of ρ = 0.2117, a sample size of 230 participants was determined. Furthermore, this sample size corresponded to 10 participants per scale item, which is commonly considered adequate for psychometric validation studies. A test-retest reliability analysis was conducted in a subsample of 77 participants, who completed the SF-LLFDI a second time after 15 days.

### Instruments

Criterion validity was evaluated by the concurrent validity approach. Disability related metrics were examined via the Barthel Index of Activities of Daily Living [28] and the Lawton Instrumental Activities of Daily Living Scale [6], whereas functional status was evaluated through the Short Physical Performance Battery [29] and the Timed Up and Go Test [30]. Construct validity was assessed by factor analysis and convergent validity. The sample’s adequacy and appropriateness for factor analysis were evaluated using Bartlett’s Test of Sphericity and the Kaiser-Meyer-Olkin (KMO) score. Convergent validity was assessed by matching SF-LLFDI results with those of the Short Form 36 Quality of Life questionnaire [31]. Furthermore, cognitive status measured by the Mini Mental State Examination (MMSE) and comorbidity burden assessed by the Charlson Comorbidity Index (CCI) were included as exploratory analyses and were not part of the primary validation framework. These measures were evaluated to explore the relationship of the Turkish SF-LLFDI with cognitive function and comorbidity burden, both of which are known to influence functional status in older adults. Pearson correlation coefficients were computed to assess the strength and direction of the correlations between SF-LLFDI and the other scales.

Sociodemographic characteristics of the participants were recorded; including sex, age, height, weight, body mass index, educational level, marital status and type of social interaction. Educational level was categorized into five groups: no formal education, primary school, high school, university and postgraduate education. Marital status was classified as either married or single. Social interaction status was grouped into three categories: living alone, living only with a spouse and living with family members.

**The Short Form–Late Life Function and Disability Instrument (SF-LLFDI)** is a comprehensive assessment tool originally developed in 2002 by Alan M. Jette and colleagues to evaluate physical functioning and disability in older adults living in the community. The original version comprises two subscales: Function and Disability. The Function component includes 32 items, while the Disability component consists of 16 items, each of which is answered twice—once for frequency and once for perceived limitation. To enhance feasibility across clinical and research settings, McAuley et al. developed a shorter version with robust psychometric properties. This abbreviated form includes 15 items for Function and 8 items for Disability [32].

The Function subscale encompasses three domains: upper extremity function, basic lower extremity function and advanced lower extremity function. The Disability subscale is divided into two domains for both frequency and limitation dimensions: performance of social roles and performance of personal roles [16]. Response options are based on a 5-point Likert scale. For the Function subscale, participants respond to the question, “How much difficulty do you have?” using the following options: 1 = Cannot do, 2 = Quite a lot, 3 = Some, 4 = A little, 5 = None.

For the Disability subscale, the frequency dimension is assessed with “How often do you do it?” (1 = Never, 2 = Almost never, 3 = Once in a while, 4 = Often, 5 = Very often), and the limitation dimension with “To what extent do you feel limited when doing it?” (1 = Completely, 2 = A lot, 3 = Somewhat, 4 = A little, 5 = Not at all).

**The Short Physical Performance Battery,** initially established in 1994 by Guralnik et al., is utilized to evaluate individuals’ physical performance and lower extremity functionality. The SPPB comprises three elements: balance ability, walking speed, and the repeated chair stand exam. Each component is evaluated on a scale from 0 to 4, yielding a maximum cumulative score of 12. Elevated scores signify superior physical performance [29,33].

**The Timed Up and Go Test**, developed by Podsiadlo et al. in 1991, evaluates balance, gait velocity, and functional ability in elderly individuals. It offers an effective method for evaluating fall risk. The test protocol requires the individual to sit in a chair, stand at command, move forward 3 meters, return to the starting position, and sit down. The duration is quantified in seconds, with reduced times signifying enhanced functional capacity [30].

**The Barthel Daily Living Activities Index,** created by Barthel and Mahoney in 1965, assesses persons’ independence in activities of daily living (ADL). Participants are evaluated based on ten fundamental activities of everyday living. Each fundamental activity of daily life is evaluated on a scale of 0, 5, 10, and 15 based on the individual’s degree of independence [28].

**The Lawton-Brody Instrumental Activities of Daily Living Scale**, created in 1969 by Dr. M. Powell Lawton and Elaine M. Brody, evaluates the degree of independence in more intricate daily living activities, especially among older persons. Participants are assessed on 8 items and obtain scores from 0 to 8 when this measure is administered. Elevated scores signify enhanced levels of functionality [6]. The ceiling effect restricts its application in individuals with elevated functional capabilities [34].

**The Mini Mental Test** was initially created in 1975 to assess cognitive processes including direction, registration memory, attention and computation, recall, and language [35]. Scoring spans from 0 to 30, and its validity and reliability have been confirmed in Turkish, with a cutoff score of 23/24 [36].

**The Short Form-36 (SF-36)** is a quality of life scale created in 1992 by Ware and Sherbourne to deliver a thorough and consistent evaluation of health state. The 36 questions facilitate the assessment of health-related quality of life across eight dimensions: physical functioning, social functioning, limitations due to physical functioning, limitations due to emotional issues, mental well-being, energy, pain, and overall health perception. Elevated scores signify superior health state [31].

**Charlson Comorbidity Index (CCI)** was utilized to determine the comorbid disease burden of the participants. This index scores 19 different medical conditions, providing a standardized measure of general health status and mortality risk [37].

Each assessment session lasted approximately 45–60 minutes and was conducted by the corresponding author in a standardized clinical setting. Data collection was performed between 1 January 2024 and 30 December 2024.

### Statistical evaluation

The gathered data were analyzed using SPSS version 23.0 (SPSS Inc., Chicago, Illinois, USA). The normality of the data distribution was evaluated by skewness and kurtosis coefficients. The following approaches were employed to assess scale reliability: test-retest reliability (Intraclass Correlation Coefficient, ICC), item-total score correlation, and Cronbach’s α coefficient. Data from the complete sample of 230 people were utilized for item-total score correlations and Cronbach’s α values. Data from the 77 participants who participated in the retest were evaluated for test-retest reliability. The evaluation of the scale’s validity involved content validity determined by expert opinion, criterion validity examined by concurrent validity methods, and structural validity analyzed via exploratory factor analysis, along with convergent validity approaches. The Kaiser-Meyer-Olkin and Bartlett tests were initially employed to assess the sufficiency and suitability of the sample size for factor analysis. Following this, exploratory factor analysis were performed.

The assessment of concurrent validity was conducted utilizing the Barthel ADL Index, Lawton IADL, Short Physical Performance Battery, and Timed Up and Go Test. The association between SF-LLFDI and SF-36 for convergent validity was assessed by computing Pearson correlation coefficients. The association between SF-LLFDI and the Mini Mental Test as well as the Charlson Comorbidity Index was assessed using Pearson correlation coefficients.

## Results

During the expert panel evaluation, some items were translated verbatim, while others were modified for cultural appropriateness. In the Function subscale, items 1, 4, 6, 9, 10, 11, 12, 13, 14, and 15 were translated employing a literal methodology. Conversely, items 2 and 5, which once used the unit “mile,” were modified to “meter” to align with the metric system employed in Turkey. Furthermore, item 3 was amended by substituting “can opener” with “vegetable corer” a tool more culturally resonant. In item 8, the term “teapot” was incorporated to enhance clarity.

In the Disability subscale, items 1, 3, 4, 5, 6, 7, and 8 were translated verbatim. Item 2, formerly denoting “restaurants or cinemas,” was modified to “park, garden, teahouse, mosque” to more accurately represent frequently used public venues in Turkish culture.

Pilot testing was conducted with 25 participants, all of whom reported complete understanding of the items. No item modifications were required following the pilot test.

A total of 230 individuals aged between 70 and 97 participated in the study, comprising 53.9% males (n = 124) and 46.1% females (n = 106). Table 1 presents the physical and sociodemographic characteristics outlined.

**Table 1:** Sociodemographic characteristics.

Physical characteristics	Mean *±* SD
Age (years)	75.33 ± 5.20
Height (cm)	163.99 ± 9.10
Weight (kg)	76.57 ± 11.56
Body mass index (kg/m2)	28.53 ± 4.22
**Sociodemographic characteristics**	
Gender	**n(%)**
Male	124(53.9)
Female	106(46.1)
**Education**	
No formal education	86(37.4)
Primary school	101(43.9)
High school	29(12.6)
University	14(6.1)
**Marital status**	
Single	67(29.1)
Married	163(70.9)
**Social interactions**	
With family	76(33)
With spouse	114(49.6)
Alone	40(17.4)

### Internal consistency

The internal consistency of the Turkish version of the SF-LLFDI was assessed using Cronbach’s α coefficient and item-total score correlation. The overall SF-LLFDI demonstrated excellent internal consistency (Cronbach’s α = 0.987, 95% CI: 0.985–0.989). Cronbach’s α coefficients for the Function, Disability–Frequency, and Disability–Limitation subscales were 0.976 (95% CI: 0.971–0.979), 0.933 (95% CI: 0.920–0.943), and 0.957 (95% CI: 0.948–0.963), respectively, indicating excellent internal consistency across all domains. The item-total score correlation analysis of the SF-LLFDI indicated that all items on the scale exhibit a strong positive correlation with the total score (p < 0.001). The item exhibiting the highest association with the overall score was “taking care of local errands” (r = 0.922, p < 0.001) within the disability subscale, whereas the item demonstrating the lowest association was “prepare meals for yourself” (r = 0.570, p < 0.001) in the same subscale. Table 2 presents the Cronbach’s α and item-total score correlation analysis for the overall assessment and subscales of the SF-LLFDI.

**Table 2:** Cronbach’s α and item-total score correlation analysis.

Subscales	Cronbach’s α	95% CI	Items	Item-Total Correlation (r)
**Total Assessment**	0.987	0.985-0.989		
**Function**	0.976	0.971-0.979		
Advanced lower extremity	0.968		Function 2	0.895**
			Function 5	0.907**
			Function 6	0.905**
			Function 10	0.915**
			Function 13	0.919**
Basic lower extremity	0.951		Function 9	0.893**
			Function 11	0.913**
			Function 12	0.889**
			Function 14	0.890**
			Function 15	0.894**
Upper extremity	0.937		Function 1	0.812**
			Function 3	0.821**
			Function 4	0.778**
			Function 7	0.905**
			Function 8	0.841**
**Disability-Frequency**	0.933	0.920-0.943		
Social role	0.857		Frequency 1	0.779**
			Frequency 2	0.875**
			Frequency 3	0.847**
			Frequency 4	0.822**
Personal role	0.912		Frequency 5	0.920**
			Frequency 6	0.572**
			Frequency 7	0.871**
			Frequency 8	0.862**
**Disability-Limitation**	0.957	0.948-0.963		
Social role	0.905		Limitation 1	0.880**
			Limitation 2	0.904**
			Limitation 3	0.901**
			Limitation 4	0.862**
Personal role	0.947		Limitation 5	0.922**
			Limitation 6	0.710**
			Limitation 7	0.885**
			Limitation 8	0.880**

### Test–retest reliability

The test-retest reliability evaluation of SF-LLFDI indicated that the scale and its subscales exhibited substantial consistency across time (p < 0.001). The ICC value for the SF-LLFDI aggregate score was determined to be 0.999.

The ICC values between 0.997 and 0.999 for all subscales and the overall score demonstrate that the test-retest reliability of the scale and its subscales is exceptional (Table 3).

**Table 3:** The ICC Values of Subscales.

Subscale	ICC	Confidence Interval	p
SF-LLFDI	0.999	[0.999–1.000]	<0.001
Function	0.999	[0.999–1.000]	<0.001
Disability–Frequency	0.997	[0.996–0.998]	<0.001
Disability–Limitation	0.998	[0.997–0.999]	<0.001

### Structural validity

Exploratory Factor Analysis (EFA) was employed to evaluate the construct validity of the scale items. The Kaiser-Meyer-Olkin (KMO) and Bartlett’s tests validated the sampling adequacy and factorability of the correlation matrices for all subscales.

The KMO scores were exceptional for the Function (0.967), Disability-Frequency (0.903), and Disability-Limitation (0.925) subscales. Bartlett’s test of sphericity was significant for each subscale: Function: χ^2^ = 9357.20, df = 105, p < 0.001; Disability-Frequency: χ^2^ = 3313.87, df = 28, p < 0.001; Disability-Limitation: χ^2^ = 4097.86, df = 28, p < 0.001, demonstrating that the data were appropriate for exploratory factor analysis.

Exploratory factor analysis was performed using principal component analysis (PCA) with Varimax rotation. PCA was selected to examine whether the translated items clustered according to the predefined domains of the original SF-LLFDI. This approach was also adopted in the Spanish validation study of the SF-LLFDI, allowing methodological consistency across language adaptations. The exploratory factor structure obtained from PCA was subsequently evaluated using confirmatory factor analysis (CFA), providing additional evidence for the structural validity of the Turkish version. All items demonstrated robust factor loadings onto their respective domains, ranging from 0.467 to 0.963, with no significant cross-loadings, confirming the conceptual clarity of the Turkish version. The cumulative variation accounted for by the three components identified in the exploratory factor analysis of the Function subscale was determined to be 83.128%. The Advanced Lower Extremity Function (5.537), had the highest eigenvalue, accounts for 32.6% of the overall variance. The Basic Lower Extremity Function (4.802) accounts for 28.27% of the overall variance, whereas the Upper Extremity Function (3.780) accounts for 22.25% of the entire variance (Table 4). The cumulative variance accounted for by the two factors derived from the exploratory factor analysis of the Disability-Frequency subscale was determined to be 79.833%. The Social Role Subscale (3.557), had the highest eigenvalue, accounts for 44.459% of the total variation. The Personal Role Subscale (2.830) accounts for 35.37% of the overall variance. The exploratory component analysis of the Disability-Limitation subscale identified two factors that accounted for 85% of the total variance. The Social Role Subscale, had the highest eigenvalue of 3.860, accounts for 48.268% of the total variance. The Personal Role Subscale (2.920) accounts for 36.73% of the overall variance (Table 5).

**Table 4:** Exploratory factor analysis of the function subscale.

Items	Rotated Factor Loadings			Subscales	Rotated Sums of Squared Loadings		
	Advanced Lower Extremity	Basic Lower Extremity	Upper Extremity		Total	Variance %	Cumulative %
**Function13**	0.838			**Advance Lower Extremity**	5.537	32.600	32.600
**Function5**	0.850						
**Function6**	0.843						
**Function2**	0.836						
**Function10**	0.836						
**Function15**		0.786		**Basic Lower Extremity**	4.802	28.272	60.872
**Function11**		0.761					
**Function12**		0.751					
**Function9**		0.702					
**Function14**		0.686					
**Function4**			0.860	**Upper Extremity**	3.780	22.255	83.128
**Function7**			0.825				
**Function8**			0.776				
**Function3**			0.725				
**Function1**			0.467				

**Table 5:** Exploratory factor analysis of the disability subscale.

Items	Rotated Factor Loadings		Subscales	Rotated Sums of Squared Loadings		
	Social Role	Personal Role		Total	Variance %	Cumulative %
**Frequency 1**	0.751		**Social Role**	3.557	44.459	44.459
**Frequency 2**	0.858					
**Frequency 3**	0.796					
**Frequency 4**	0.819					
**Frequency 5**		0.914	**Personal Role**	2.830	35.373	79.833
**Frequency 6**		0.863				
**Frequency 7**		0.713				
**Frequency 8**		0.963				
**Limitation 1**	0.750		**Social Role**	3.860	48.268	48.268
**Limitation 2**	0.778					
**Limitation 3**	0.819					
**Limitation 4**	0.788					
**Limitation 5**		0.753	**Personal Role**	2.920	36.733	85.002
**Limitation 6**		0.913				
**Limitation 7**		0.816				
**Limitation 8**		0.738				

Following the exploratory analysis, Confirmatory Factor Analysis (CFA) was conducted to verify the model. To optimize model fit, error terms were correlated between a limited number of theoretically related item pairs. Specifically, in the Function subscale, items 2 (‘running 800 meters’) and 13 (‘climbing stairs while carrying a load’) share substantial conceptual overlap, as both require high-intensity cardiovascular effort and maximal lower extremity strength. In the Disability subscale, items 6 (‘preparing meals’) and 8 (‘attending to personal care’) are similarly related, as both represent home-based independence activities requiring comparable fine motor skills and standing tolerance. These modifications were guided by AMOS modification indices in conjunction with theoretical rationale rather than by data-driven decisions alone, and the number of correlated error terms was kept to a minimum to avoid overfitting [38]. No alternative model specifications were systematically tested; the decision to correlate error terms was based on theoretical rationale and modification indices rather than model comparison. Following these modifications, the resulting model fit indices χ^2^/ df = 2.76–4.71; RMSEA = 0.069–0.078; CFI = 0.960–0.984; GFI = 0.900–0.947) demonstrated that the structural model representing the functional and disability profile of the Turkish elderly population was statistically validated and achieved a high level of fit with the observed data (Table 6) [39]. These indices fall within the acceptable thresholds established in the psychometric literature for scale adaptation studies [40,41]. Path diagrams illustrating the final CFA models for the Function and Disability subscales, including the correlated residual terms, are presented in S1 Fig and S2 Fig.

**Table 6:** Model fit indices from Confirmatory Factor Analysis (CFA).

Fit Index	Value	Threshold	Interpretation
**χ2/df**	2.76	≤ 5.0	Acceptable fit
**RMSEA**	0.078	≤ 0.08	Acceptable fit
**CFI**	0.96	≥ 0.90	Acceptable fit
**GFI**	0.90	≥ 0.90	Acceptable fit

### Convergent validity

A strong correlation exists between the SF-LLFDI function subscale and the SF-36 physical functioning subscale (r = 0.938, p < 0.001). Additionally, a strong positive correlation is observed between the disability-limitation subscale of SF-LLFDI and the physical limitation subscale of SF-36 (r = 0.879, p < 0.001). Furthermore, substantial positive correlations were identified between the SF-LLFDI and the SF-36 mental well-being subscale (r = 0.664, p < 0.001).

### Concurrent validity

Consequently, SF-LLFDI exhibited a substantial positive correlation with Barthel ADL Index (r = 0.960, p < 0.01), Lawton IADL (r = 0.937, p < 0.01) and SPPB (r = 0.986, p < 0.01); whereas SF-LLFDI and TUG demonstrated a significant negative correlation (r = −0.975, p < 0.01). Upon assessment of subscale, the SF-LLFDI functional subscale exhibited a strong positive association with SPPB (r = 0.986, p < 0.001), whereas a highly significant negative correlation was identified between the SF-LLFDI functional subscale and TUG (r = −0.970, p < 0.001). A strong association exists between the disability-frequency subscale of SF-LLFDI and Barthel Index (r = 0.948, p < 0.001) as well as Lawton IADL (r = 0.928, p < 0.001).

A strong association exists between the disability-limitation subscale of the SF-LLFDI and the Barthel Index (r = 0.951, p < 0.001) as well as the Lawton IADL (r = 0.927, p < 0.001). The SF-LLFDI and its subscales, when assessed alongside the aforementioned scales, demonstrate significant correlations that align with expectations, so affirming the concurrent validity of the scale.

### Correlations with other measures

SF-LLFDI total scores correlated negatively with the Charlson Comorbidity Index (r = −0.897, p < 0.001), suggesting higher comorbidity is associated with lower functional status. A strong positive correlation was found between the SF-LLFDI total and Mini Mental State Examination scores (r = 0.848, p < 0.001), indicating an association between cognitive and functional status. Although the MMSE served as an inclusion criterion to ensure cognitive capacity for self-reporting, its inclusion in correlation analyses was theoretically motivated: cognitive status is a well-established determinant of functional independence in older adults, and examining this association within a cognitively intact sample provides clinically meaningful information about the relationship between cognition and self-reported function. Similarly, the Charlson Comorbidity Index was included to explore the association between chronic disease burden and functional status, consistent with evidence that multimorbidity is a primary driver of disability in aging populations [47]. Male participants exhibited significantly higher scores than female participants across all subscales and the overall score (p < 0.001). This study revealed that married participants had considerably higher scores than their unmarried counterparts across all subscales and the overall score (p < 0.001). According to the analyses performed, no participant attained either the lowest or maximum score on the SF-LLFDI or its subscales. Consequently, neither floor nor ceiling effects were observed in the SF-LLFDI or its subscales, as 0% of participants achieved the minimum or maximum possible scores.

## Discussion

This study aimed to translate, culturally adapt and evaluate the reliability and validity of the Turkish version of the Short Form-Late Life Function and Disability Instrument in a population of older adults. The findings revealed that the Turkish adaptation demonstrates excellent psychometric properties, confirming its utility as a culturally appropriate tool to assess function and disability in the geriatric population. The Late-Life Function and Disability Instrument (LLFDI) was originally developed in English to assess physical functioning and disability among older adults and has since been translated into various languages, Swedish [13], Hebrew [14], Korean [15], Dutch [16] and Arabic [17], while validity and reliability studies of the SF-LLFDI have been conducted in Spanish [18] and German [19].

The scale was culturally adapted for the Turkish context following established guidelines, with item-level modifications to ensure semantic, idiomatic, and conceptual equivalence; detailed translation outcomes are presented in the results section.

Additionally, the distribution of participants in terms of educational level and marital status appeared consistent with findings reported in the existing literature. The clearly defined inclusion and exclusion criteria contributed to a well-characterized and internally consistent study population. In this regard, the study was conducted within a clearly defined methodological framework. Male participants exhibited significantly higher scores than female participants across all subscales and the overall score (p < 0.001). This data aligns with the disability process model posited by Verbrugge and Jette, suggesting that disability is more prevalent among older women [42]. A previous meta-analysis demonstrated that age and gender are significant determinants of physical functioning and disability, with advanced age and female sex identified as facilitating factors for disability. Age-related loss of muscle strength, chronic disease burden, and social isolation were proposed as contributing mechanisms. The disadvantaged position of women in the disability process was attributed to higher prevalence of chronic conditions, longer life expectancy, and the impact of gender roles [43]. The finding that married participants scored significantly higher across all domains aligns with the broader literature suggesting that marital status plays a protective role in the context of disability. A previous study reported that marriage serves as an important source of support in coping with physical and emotional challenges, and reduces the perceived burden associated with disability-related difficulties [44].

For the reliability analysis of the Turkish version of the SF-LLFDI, Cronbach’s alpha coefficients, item-total correlations, and test-retest reliability methods were employed. In this respect, the Turkish adaptation appears to offer a more comprehensive reliability assessment compared to other language versions. In the literature, a Cronbach’s alpha value of 0.70 is generally accepted as the threshold for adequate internal consistency [45]. In the present study, Cronbach’s alpha coefficients for both the overall scale and its subscales exceeded this threshold. These findings are comparable to those reported in the Spanish adaptation [18] and higher than those reported in some of the other language versions.

In the item-total correlation analysis, all items across the subscales demonstrated significant and positive correlations with the total score, with correlation coefficients exceeding r = 0.572 (p < 0.001). These findings indicate that each item meaningfully contributed to the overall score, reflecting a high level of internal consistency at the item level. In the Turkish version of the SF-LLFDI, item-total correlation analysis revealed statistically significant and strong positive associations across all subscales (r > 0.572; p < 0.001). In contrast, such item-level reliability analysis was not reported in the original SF-LLFDI study or in its German [19] and abbreviated version [32]. This indicates that the Turkish validation study provided a more detailed psychometric assessment at the item level and strengthened the evidence for internal consistency.

Reliability analyses revealed that the Turkish version of the SF-LLFDI demonstrated exceptionally high temporal stability (ICC: 0.997–0.999). These findings are consistent with the high temporal stability observed in other language versions of the SF-LLFDI. Indeed, the ICC value of 0.982 reported for the Spanish version [18] and the item-level ICC values reaching 0.999 in the Turkish version of LLFDI [12] confirm that both forms of the instrument yield remarkably consistent results in Turkish and other populations. These high reliability levels can be attributed to the methodological standards and sample characteristics of our study; notably, community-dwelling older adults with intact cognition and preserved function are unlikely to experience meaningful changes in functional status over a 15-day interval in the absence of acute health events, which mathematically drives ICC values toward the upper limit. To minimize the risk of recall bias, participants were not informed of their initial responses at the time of the second assessment, and all assessments were conducted by the same researcher under identical conditions in the same clinical setting. The clustering of scores at the high end of the scale among our community-dwelling, functionally independent, and healthy elderly participants restricted the score variance and mathematically drove the ICC values toward the upper limit. Furthermore, performing assessments by the same experienced researcher under standardized conditions and the high cognitive status of the participants (MMSE  > 24) contributed to achieving this high level of stability by minimizing measurement error. Although the internal consistency coefficients were exceptionally high, these findings should be interpreted with caution. Cronbach’s α values approaching 1.00 may partly reflect the assessment of closely related functional and disability domains, resulting in conceptually related item responses. In addition, the relatively homogeneous characteristics of our study population, consisting of community-dwelling older adults with preserved cognitive function and relatively high functional capacity, may have restricted score variability. Reduced variability is known to mathematically increase both internal consistency and test–retest reliability estimates. Therefore, the exceptionally high Cronbach’s α and ICC values observed in this study are likely attributable to both the conceptual coherence of the SF-LLFDI and the characteristics of the study sample, rather than indicating unnecessary redundancy among items.

The findings of the exploratory factor analysis provide robust evidence for the construct validity of the Turkish version of the SF-LLFDI. The EFA solutions, which aligned with the original conceptual structure of the instrument, explained a substantial proportion of the variance (83.13% for Function, 79.83% for Disability–Frequency, and 85.00% for Disability–Limitation). The KMO values (0.903–0.967) and the significance of Bartlett’s test confirmed the adequacy of the sample and the suitability of the data for factor analysis. Furthermore, the strong factor loadings observed (ranging between 0.467 and 0.963) without any problematic cross-loadings indicate that the item structure was clearly defined and consistent with the theoretical framework. These results collectively support the structural integrity of the scale and suggest that the Turkish version successfully preserves the dimensional configuration of the original instrument. The psychometric findings of the present study are generally consistent with those reported in previous cross-cultural adaptations of the SF-LLFDI. The Spanish validation demonstrated excellent internal consistency (Cronbach’s α = 0.974) and test–retest reliability (ICC = 0.982–0.989), while the German version also reported good internal consistency (Cronbach’s α = 0.78–0.89) and reliability (ICC = 0.81–0.96). Although the reliability coefficients observed in the present study were slightly higher, the overall psychometric performance and construct validity of the Turkish version were comparable with these previous adaptations, supporting the cross-cultural robustness of the instrument. The somewhat higher reliability estimates in our study may be explained by the relatively homogeneous characteristics of our study population, as discussed above. A similar factorial structure was reported in the Spanish validation study [18] although the total variance explained in that version was lower (67.2%), indicating a potentially higher construct clarity in the Turkish adaptation.

In this study, the concurrent validity of the Turkish version of the SF-LLFDI was assessed using widely accepted gold-standard instruments in the domains of function and disability. Strong and statistically significant correlations were observed between the SF-LLFDI total score and the Barthel Index (r = 0.960), Lawton IADL (r = 0.937), SPPB (r = 0.986), and TUG test (r = –0.975). Subscale analyses further supported these findings, with the Function component showing high correlations with the SPPB (r = 0.986) and TUG (r = –0.975), and the Disability–Frequency and Disability–Limitation components exhibiting strong associations with both the Barthel Index and Lawton IADL (ranging from r = 0.927 to r = 0.951). These values were considerably higher than those reported in previous validation studies. For instance, the Spanish version reported correlations of r = 0.827 with the Barthel Index and r = 0.693 with the Lawton IADL, while the German study found only r = 0.36 between the Function component and the Barthel Index. These results suggest that the Turkish version of the SF-LLFDI demonstrates excellent concurrent validity and robust convergence with established functional and disability measures. Although the high magnitude of these correlations might suggest redundancy between instruments, it is important to note that the SF-LLFDI and the performance-based measures capture distinct aspects of function. The SF-LLFDI reflects the patient’s subjective perception of functional difficulty and disability in social and personal roles, whereas measures such as the SPPB and TUG assess objective physical performance under standardized conditions. The strong correlations observed in this study are therefore indicative of excellent convergent validity rather than redundancy, and are consistent with findings in community-dwelling older adults with preserved function, where self-reported and observed performance tend to align closely.

The convergent validity of the Turkish version of the SF-LLFDI was supported through its strong associations with theoretically related constructs such as quality of life, cognitive status, and comorbidity burden. Notably, the SF-LLFDI total score demonstrated strong positive correlations with the SF-36 overall score (r = 0.868, p < 0.001), Physical Function (r = 0.938), and Physical Role Limitation (r = 0.887) subscales. These values are consistent with, and in some cases exceed, those reported in previous studies (e.g., Dubuc et al., r = 0.85). In addition, significant correlations were observed with widely accepted ADL measures—Barthel Index (r = 0.960) and Lawton IADL Scale (r = 0.937)—further affirming the scale’s external validity. Moderate-to-strong correlations with mental health (SF-36 Mental Well-being, r = 0.664) and cognitive function (MMSE, r = 0.848) also suggest a meaningful link between perceived function and psychological-cognitive status, aligning with findings from a study reported lower LLFDI scores among individuals with higher depression severity [46]. A strong negative correlation with the Charlson Comorbidity Index (r = –0.897) further supports known associations between multimorbidity and reduced physical function, in line with evidence presented by a previous study [47]. Collectively, these findings demonstrate that the Turkish version of the SF-LLFDI exhibits strong convergent validity across physical, psychological, and clinical domains in older adults.

The findings of this study demonstrate that the Turkish version of the SF-LLFDI exhibits high internal consistency, excellent test–retest reliability, and strong structural and concurrent validity. The scale showed robust correlations with theoretically related constructs, further confirming its convergent validity. Accordingly, the Turkish SF-LLFDI can be considered a valid and reliable instrument for assessing function and disability in older adults, suitable for both clinical practice and research purposes.

Key strengths of the study include the carefully defined inclusion and exclusion criteria, which resulted in a well-characterized study population, the strong psychometric performance of the scale despite the low educational background of the participants; and the consistent, face-to-face administration by a trained physician. Furthermore, the use of multiple psychometric approaches for reliability assessment, and the comprehensive validation strategy involving well-established instruments for each domain (e.g., SPPB and TUG for function), enhance the methodological rigor of the study.

Nevertheless, this study has some limitations. The deliberate exclusion of individuals with severe neurological or orthopedic disorders limiting participation, together with the inclusion of only community-dwelling older adults with preserved cognitive function, resulted in a relatively homogeneous sample with higher functional status. This may have contributed to the elevated reliability coefficients and strong correlation values observed. Therefore, the findings may not be directly generalizable to older adults with greater functional impairment, cognitive decline, or those living in institutional care settings. In addition, responsiveness to clinical change was not evaluated in the present study. Future longitudinal studies are warranted to determine the ability of the Turkish SF-LLFDI to detect changes over time. Future studies should further evaluate the psychometric properties of the Turkish SF-LLFDI in more heterogeneous elderly populations. Only a small proportion of participants had completed secondary or higher education, which may limit the generalizability of findings to populations with higher socioeconomic or educational backgrounds. Moreover, all participants were community-dwelling older adults, and no individuals from institutional care settings such as nursing homes were included. As functional and disability profiles in institutionalized populations may differ substantially from those living independently, future studies should validate the Turkish SF-LLFDI in more diverse settings and populations, including those with lower functional capacity, higher comorbidity burden, and varying levels of cognitive impairment.

## Conclusion

The Turkish version of the SF-LLFDI demonstrated excellent internal consistency (Cronbach’s α = 0.987), outstanding test–retest reliability (ICC = 0.997–0.999), and robust structural, convergent, and concurrent validity in community-dwelling older adults aged 70 years and above. The absence of floor or ceiling effects further supports its sensitivity across the functional spectrum. The Turkish SF-LLFDI is therefore recommended as a valid and reliable assessment tool for both clinical practice and research in geriatric populations.

## Supporting information

S1 Data Anonymized English-language dataset underlying the findings of this study. (XLSX)

S1 Fig CFA path diagram for the Function subscale. ALEF = Advanced Lower Extremity Function; BLEF = Basic Lower Extremity Function; UEF = Upper Extremity Function. Values on single-headed arrows represent standardized factor loadings; values adjacent to error circles represent error variances. The curved double-headed arrow between e1 (M2, ‘running 800 meters’) and e5 (M13, ‘climbing stairs while carrying a load’) indicates a correlated residual (r = .39), justified by the shared cardiovascular and lower extremity demands of these items.
(TIF)

S2 Fig CFA path diagram for the Disability subscale. SR = Social Role; PR = Personal Role. The curved double-headed arrow between e6 (M6, ‘preparing meals’) and e8 (M8, ‘attending to personal care’) indicates a correlated residual, reflecting the shared fine motor and standing tolerance demands of these home-based independence activities.
(TIF)

S1 Appendix Turkish version of the Short Form-Late Life Function and Disability Instrument (SF-LLFDI). (PDF)
