# Supplementary material for: Validity and reliability of the short form-late life function and disability instrument Turkish version
Source: PLoS One. 2026 Nov 10;21(11):e0361134. doi: 10.1371/journal.pone.0361134 (PMC13750544; doi:10.1371/journal.pone.0361134)
Supplement: Supplementary file 1 [file pone.0361134.s004.pdf]

Kısa Form-Geç Yaşam İşlev ve Engellilik Aracı Türkçe Versiyonu

	İşlev Soruları	*Ne kadar zorlanırsınız ?*				
		Hiç	*Biraz*	*Bazen*	*Oldukça fazla*	*Yapamam*
1	*Daha önce açılmamış bir kavanozun kapağını herhangi bir alet kullanmadan açma*	5	4	3	2	1
2	*800 metre veya daha fazla koşma*					
3	*Yemek hazırlamak için yaygın olarak kullanılan araçları kullanma (örneğin; kabak oyacağı, patates soyacağı veya keskin bıçak)*					
4	*Bir elinde dolu bir bardak su tutma*					
5	*Gerektiğinde dinlenerek 1.5 kilometre yürüyüş yapma*					
6	*Korkuluk kullanmadan dışarıda bir merdiven çıkıp inme*					
7	*Yalnızca ellerinizi kullanarak bir atıştırmalık yiyecek (örneğin; kraker, bisküvi, gofret) paketini açma*					
8	*Büyük bir sürahiden (çaydaklıktan) bardağa su dökme*					
9	*(Sedan) bir araba/taksiye binme ve inme*					
10	*İçeride tırabzan kullanarak üç kat merdiven çıkıp inme*					
11	*Temizlik yapmak için mutfak sandalyesini kaldırıp taşıma*					
12	*Yüksek bir dolaba ulaşmak için bir tabure kullanma*					
13	*Bir şeyi iki elde taşıyarak bir merdiven çıkma (örneğin, çamaşır sepeti)*					
14	*Ayakta dururken yerden bir parça giysi almak için eğilme*					
15	*Birkaç apartman bloğu yürüme*					

	Engellilik Soruları	*Ne sıklıkla yaparsınız ?*					*Yaparken kendinizi ne kadar kısıtlanmış hissedersiniz?*				
		Çok sık	*Sıkça*	*Arada bir*	*Neredeyse hiç*	*Asla*	*Hiç*	*Biraz*	*Bazen*	*Çok*	*Tamamen*
		5	4	3	2	1	5	4	3	2	1
1	*Evine yemek veya eğlence için insanları davet etme*										
2	*Başkalarıyla birlikte halka açık yerlere (park, bahçe, kıraathane, cami gibi) gitme*										
3	*Arkadaşları ve aileyi evlerinde ziyaret etme*										
4	*Şehir dışına en az bir gece konaklamak üzere seyahat etme*										
5	*Yerel işlerle ilgilenme. (Bu, yiyecek ve kişisel eşyalar için alışveriş yapmak amacıyla market veya pazara gitmeyi ve sorumluluk almayı ve bankaya gitmeyi içerebilir)*										
6	*Kendiniz için yemek hazırlama (Buna planlama, yemek pişirme, servis yapma ve temizlik dahildir)*										
7	*Ev işleri ve finansmanla ilgilenme (Bu, paranızı yönetmek ve sorumluluk almak, faturaları ödemek, ev sahipleri veya kiracılarla uğraşmak, kamu hizmetleri şirketleri veya devlet kurumları ile ilgilenmek gibi görevleri içerebilir)*										
8	*Kendi kişisel bakım ihtiyaçlarına özen gösterme. (Bu, banyo yapma, giyinme ve tuvalet ihtiyaçlarını karşılama gibi şeyleri içerir)*										
